# Prominent Neuroprotective Potential of Indole-2-*N*-methylpropargylamine: High Affinity and Irreversible Inhibition Efficiency towards Monoamine Oxidase B Revealed by Computational Scaffold Analysis

**DOI:** 10.3390/ph17101292

**Published:** 2024-09-28

**Authors:** Lucija Vrban, Robert Vianello

**Affiliations:** Laboratory for the Computational Design and Synthesis of Functional Materials, Division of Organic Chemistry and Biochemistry, Ruđer Bošković Institute, 10000 Zagreb, Croatia; lucija.vrban@irb.hr

**Keywords:** irreversible inactivation, covalent drugs, monoamine oxidase enzyme, neurotransmitters, hydride transfer, neurodegeneration, antiparkinsonian drugs, oxidative stress

## Abstract

**Background**: Monoamine oxidases (MAO) are flavoenzymes that metabolize a range of brain neurotransmitters, whose dysregulation is closely associated with the development of various neurological disorders. This is why MAOs have been the central target in pharmacological interventions for neurodegeneration for more than 60 years. Still, existing drugs only address symptoms and not the cause of the disease, which underlines the need to develop more efficient inhibitors without adverse effects. **Methods**: Our drug design strategy relied on docking 25 organic scaffolds to MAO-B, which were extracted from the ChEMBL20 database with the highest cumulative counts of unique member compounds and bioactivity assays. The most promising candidates were substituted with the inactivating propargylamine group, while further affinity adjustment was made by its N-methylation. A total of 46 propargylamines were submitted to the docking and molecular dynamics simulations, while the best binders underwent mechanistic DFT analysis that confirmed the hydride abstraction mechanism of the covalent inhibition reaction. **Results**: We identified indole-2-propargylamine **4fH** and indole-2-*N*-methylpropargylamine **4fMe** as superior MAO-B binders over the clinical drugs rasagiline and selegiline. DFT calculations highlighted **4fMe** as more potent over selegiline, evident in a reduced kinetic requirement (ΔΔ*G*^‡^ = −2.5 kcal mol^−1^) and an improved reaction exergonicity (ΔΔ*G*_R_ = −4.3 kcal mol^−1^), together with its higher binding affinity, consistently determined by docking (ΔΔ*G*_BIND_ = −0.1 kcal mol^−1^) and MM-PBSA analysis (ΔΔ*G*_BIND_ = −1.5 kcal mol^−1^). **Conclusions**: Our findings strongly advocate **4fMe** as an excellent drug candidate, whose synthesis and biological evaluation are highly recommended. Also, our results reveal the structural determinants that influenced the affinity and inhibition rates that should cooperate when designing further MAO inhibitors, which are of utmost significance and urgency with the increasing prevalence of brain diseases.

## 1. Introduction

Monoamine oxidases (MAOs) are a family of flavoenzymes responsible for the metabolism of a wide range of endogenous and exogenous amines, in particular, monoamine neurotransmitters, such as dopamine, serotonin, and noradrenaline [1]. The two enzyme isoforms, monoamine oxidase A (MAO-A) and monoamine oxidase B (MAO-B), differ in substrate preference, inhibitor specificity, and distribution in cells and tissues [2]. MAO enzymes are more common inside neurons and astroglial cells as part of the central nervous system, but are distributed in other organs throughout the human body as well [3]. MAO-A is mainly found in the gastrointestinal tract, lung, liver, and placenta, while MAO-B is preferentially expressed in platelets and accounts for over 80% of this enzyme within the brain [4,5]. Extensive crystallographic studies revealed that MAO-A hosts a large monopartite active site cavity, allowing it to prefer bulkier substrates such as serotonin, adrenaline, and noradrenaline [6,7]. In contrast, the MAO-B cavity is bipartite and comprises two separate areas, the substrate cavity and the entrance cavity, which can adopt two different arrangements (“open” and “closed”) depending on the conformation of the side chain of the “gating” Ile199 residue [8,9]. Because of that, MAO-B primarily oxidizes smaller phenylethylamine and benzylamine, while dopamine, tyramine, and tryptamine are equally efficiently metabolized by both isoforms [10].

**Scheme 1:**
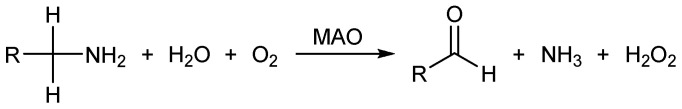
The overall amine conversion catalyzed by MAO enzymes.

Given their role in neurotransmitter degradation, MAOs are well-known pharmacological targets in brain disorders [11]. Several MAO inhibitors (MAOI) are clinically approved for the treatment of major depressive disorder and Parkinson’s disease, as they promote an increase in monoamine neurotransmitter levels [2]. In addition, the therapeutic potential of MAOI appears to be even more valuable, since an over-activity of MAOs can lead to an elevated production of harmful species, such as aldehydes and hydrogen peroxide [12]. The latter are byproducts of MAO catalytic activity that relies on the rate-limiting hydride transfer from the C_α_–H moiety vicinal to the amino group onto the N5 atom of the FAD co-factor [13,14,15], which has been confirmed through a series of experiments [16,17,18] and computations [19,20,21,22] on MAO and other members of a large family of flavoenzymes. This is followed by the water-assisted amine proton transfer onto the N1 atom on FAD, which offers a fully reduced FADH_2_ and releases a neutral imine that is non-enzymatically hydrolyzed into the final aldehyde. Lastly, the enzyme is regenerated with the molecular O_2_ that restores the oxidized FAD and liberates H_2_O_2_ (Scheme 1) [10]. With this in mind, the overexpression of MAO and upregulation of its activity, which has been found in age-associated diseases, was suggested to contribute to the development of oxidative and inflammatory stress. Moreover, MAOs have recently been found to be highly expressed in different types of cancers, which is opening new frontiers for MAOIs as potential antiproliferative agents as well [23,24,25].

On the other hand, older age is one of the risk factors for the incidence of many, if not all, neurodegenerative disorders. Given that the proportion of the elderly population is constantly increasing due to improved living conditions and more accessible public healthcare, neurodegenerative diseases were the fifth leading global cause of death in 2016. Every three seconds, someone in the world develops dementia, and in 2020, more than 55 million people were diagnosed with it. The World Health Organization estimates that these numbers will double every 20 years, resulting in 78 million affected individuals in 2030 and 139 million in 2050 [26]. These alarming facts indicate the necessity for the development of new drugs that target the cause or at least the symptoms of neurodegeneration. Additionally, recent studies suggest that the neurological symptoms that accompany the SARS-CoV-2 infection have a basis in neurotransmitter metabolism disorders [27]. Emerging evidence has suggested that the SARS-CoV-2 virus may activate microglial cells and play a role in triggering Parkinson’s disease as well as other neurological diseases [28,29].

**Figure 1:**
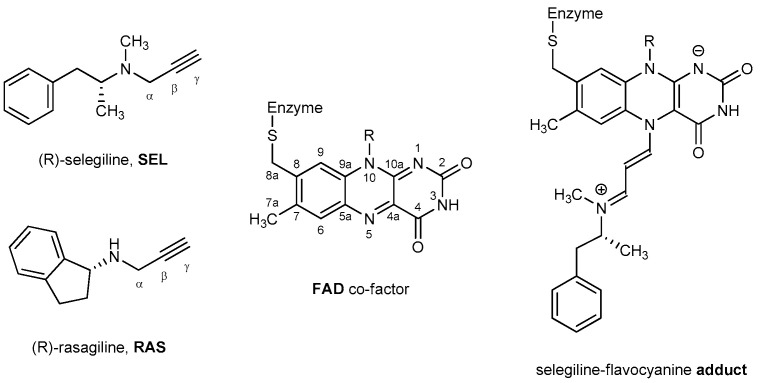
The chemical structures of systems discussed here, together with the relevant atom numbering.

The development of MAO inhibitors was undertaken with the nonselective reversible inhibitors iproniazid and phenelzine [30], yet these quickly revealed diverse adverse effects, including the so-called “cheese reaction”, involving severe, potentially lethal hypertensive crises following the consumption of foods rich in tyramine [31]. Since tyramine oxidation occurs exclusively by intestinal MAO-A, this fact prompted researchers to characterize selective MAO-B inhibitors. These were demonstrated to be effective treatment options, whilst being free from this potential interaction, which warrants their use without the restriction of a low-tyramine diet [32]. Inhibiting MAO-B not only prolongs the half-life of dopamine and extends its neurotransmission effect for relieving motor symptoms, it also prevents the MAO-B-mediated oxidative damages during dopamine degradation [33], which motivated us to focus our work on this isoform. In this context, MAO-B has been the main target for the treatment of many neurodegenerative disorders such as Parkinson’s and Alzheimer’s disease since the 1960s [34]. During this time, several drugs have been developed, where the majority of them are mechanism-based irreversible inhibitors whose activity relies on forming a covalent bond with the enzyme (Figure 1), which is the most successful way of inhibiting MAO-B in vivo [35,36]. Among them, selegiline (**SEL**) and rasagiline (**RAS**), which became commercially available in the USA in 1996 and 2006, respectively (Figure 1), proved to be efficacious for the PD treatment, while being administered without the restriction of a low-tyramine diet [32]. **SEL** is metabolized in the body via the cytochrome P450 2B6/2C19 to *L*-methamphetamine, then to *L*-amphetamine, which inhibits dopamine transport to vesicles, leading to dopamine auto-oxidation in the cytoplasm and an additional source of reactive oxygen species. Due to its amphetamine metabolite, **SEL** causes insomnia, decreased appetite, restlessness, and loss of selectivity at higher doses. On the other hand, **RAS** is metabolized via the cytochrome P450 1A2 into 1-aminoindan, which also acts as a neuroprotective [37] and has similar side effects to **SEL**, but much less intense. However, its binding is not as pharmacodynamically favorable as that of **SEL**. In high doses, it inhibits both MAO-A and MAO-B, so dietary restrictions are still required when taking these drugs [38]. Therefore, both anti-MAO-B drugs are still linked with significant untoward effects [39] and treat only the symptoms of a disease, not the cause, which underlines the necessity for the design of new and more potent compounds.

Most antiparkinsonian drug designs start with the structure of the previously mentioned drugs and then advance to the modification of the heteroaromatic rings and/or adding of other moieties. The furtherance of computer science [40,41,42] potentiates the use of chemical structure databases for screening an immense number of molecules against specific biological targets with known structures which, arguably, require little to no expertise of the targeted system, and still proved to be ineffectual in the case of MAO-B. Time is pressing for a change in the drug design perspective since no other drug-developing stratagems have yielded more potent and safer drugs in the last few decades. With this in mind, great interest remains in the development of new drugs, novel delivery systems, and drug combinations, which are of utmost importance with the growing prevalence of brain illnesses [43]. MAO-B inhibitors are still widely used in the management of Parkinson’s and Alzheimer’s diseases and a variety of other psychiatric disorders and continue to be investigated for their therapeutic value and disease-modifying potential [44,45,46,47,48].

To address this issue, we utilized an approach that relies on 20 organic scaffolds that Zdrazil and Guha found in the ChEMBL20 database to have the highest cumulative counts of unique member compounds and bioactivity assays [49]. Promising candidates, as elucidated by docking simulations, were joined with the propargylamine core, which is responsible for the MAO-B inhibition mechanism with both **SEL** and **RAS** (Figure 1), but are also highly relevant for drug discovery in general [50,51,52]. Afterwards, fine-tuning of the activity was probed by exchanging N–CH_3_ and N–H propargylamine groups, which we earlier showed can lead to improvements in MAO inactivation efficiency [53,54] and selectivity [55]. To investigate the target potency, molecular docking was performed on the newly designed candidate molecules. Those that successfully docked inside the aromatic cage in a proper orientation and higher affinities over reference drugs were subjected to classical molecular dynamics (MD) simulations. Later, candidates with thermodynamic profiles more favorable than **RAS** and **SEL** were further evaluated by the Molecular Mechanics/Poisson–Boltzmann Surface Area method (MM-PBSA) to estimate the binding free energy and to unravel which amino acids have the largest impact on the affinity. Lastly, a selected number of identified candidates were submitted to the mechanistic DFT calculations using a truncated cluster model of the enzyme active site, while an NBO population analysis was performed to confirm the inactivation of the hydride abstraction mechanism.

## 2. Results and Discussion

### 2.1. Docking of Organic Scaffolds into the MAO-B Active Site

**Figure 2:**
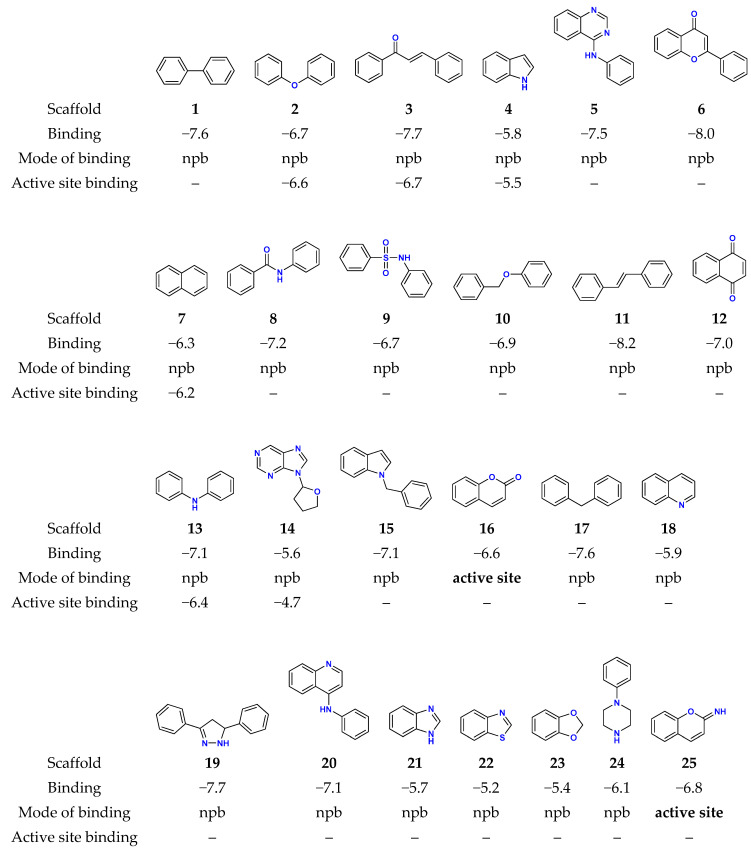
The structures of the investigated organic scaffolds, together with their most favorable MAO-B binding modes and affinities (in kcal mol^−1^), as obtained with the docking simulations. For some of the scaffolds with the preferred nonproductive binding (npb), the affinity corresponding to the less favorable active site binding is also provided.

The starting point of our analysis were organic scaffolds **1**–**20** identified by Zdrazil and Guha to have the highest numbers of unique compounds (≥200) reported in the ChEMBL20 database from 1998 to 2014 [49]. These are depicted in Figure 2 together with benzimidazole (**21**), benzothiazole (**22**), 1,3-benzodioxole (**23**), *N*-phenylpiperazine (**24**), and iminocoumarin (**25**), which are selected based on our broad experience in their derivatization towards compounds with useful photophysical [56], antioxidant [57], and antitumor properties [58].

**Figure 3:**
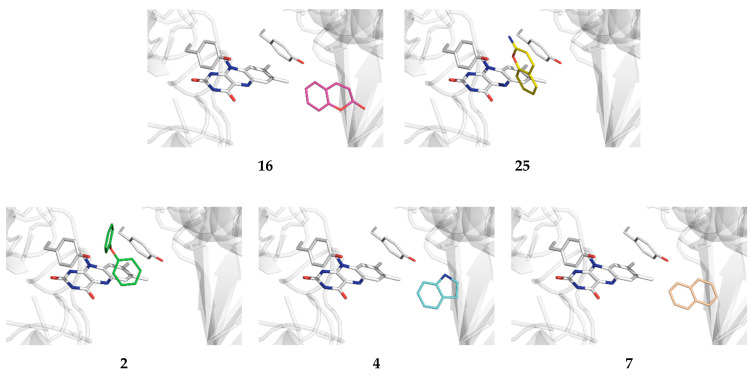
The binding position of the selected organic scaffolds within the MAO-B active site identified through docking simulations.

Systems **1**–**25** were submitted to the docking analysis in order to probe their MAO-B affinities and identify the most promising scaffolds for the next steps of the analysis. The data in Figure 2 show that all of the scaffolds are found within a narrow range of affinities, spanning values from −5.2 kcal mol^−1^ for benzothiazole **22** to −8.2 kcal mol^−1^ for stilbene **11**. However, a large number of the considered skeletons do not bind to the active site, but most preferably attach to other nonproductive positions on the enzyme surface, which limits their usefulness. Still, notable exceptions are provided by coumarin (**16**) and iminocoumarin (**25**), whose active site affinities are between −6.6 and −6.8 kcal mol^−1^, respectively, thus highlighting their significance for our drug design strategy (Figure 3). This conclusion is additionally confirmed by a range of coumarin [59,60] and iminocoumarin [61,62] derivatives identified in the literature as potent MAO-B inhibitors.

Also, a detailed inspection of all of the binding poses among systems most preferably attaching to nonproductive positions reveals that some scaffolds do bind to the enzyme active site, with only marginally lower affinities. This still allows for their significant population within the druggable area and filters them as useful candidates as well. The latter holds for diphenylether (**2**), indole (**4**), and naphthalene (**7**), whose placement within the MAO-B active site is between only 0.1 and 0.3 kcal mol^−1^ less exergonic over the identified most potent pose (Figure 3). Such an approach is justified by realizing that an analogous docking protocol for propylbenzene, the matching scaffold of a potent clinical inhibitor **SEL** (Figure 1), revealed that it binds MAO-B in a nonproductive way with the affinity of −5.8 kcal mol^−1^, while its active site placement is only marginally lower at −5.6 kcal mol^−1^.

### 2.2. Docking of Propargylamine Derivatives into the MAO-B Active Site

**Figure 4:**
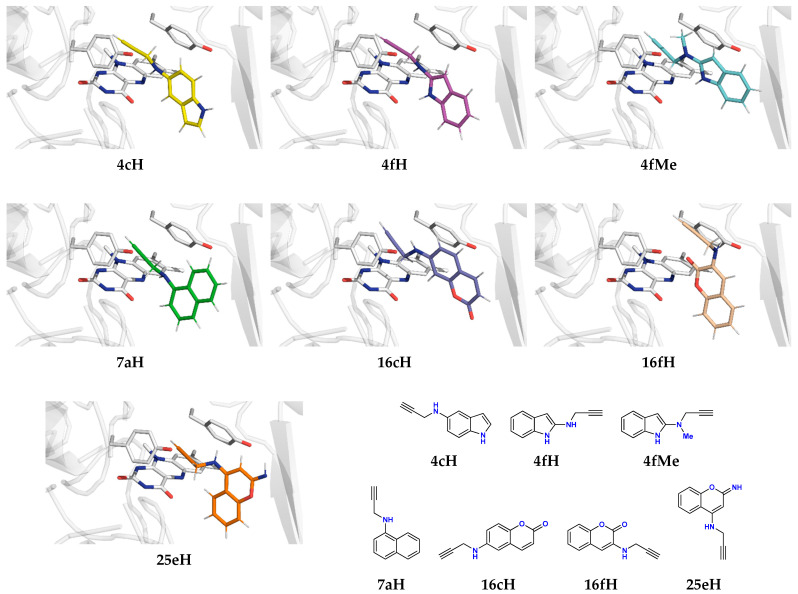
The structure of potent propargylamine inhibitors **4cH**, **4fH**, **4fMe**, **7aH**, **16cH**, **16fH**, and **25eH** and their binding position within the MAO-B active site relative to the FAD co-factor and “aromatic cage” Tyr398 and Tyr435 residues, as elucidated from the docking analysis. These complexes were carried to the molecular dynamics simulations and the subsequent DFT calculations.

After the preceding docking of pure organic scaffolds identified five promising candidates, **2**, **4**, **7**, **16**, and **25**, we proceeded by designing their propargylamine derivatives with a direct C–N(propargylamine) bond at all chemically non-equivalent positions over the scaffold’s structure. In doing so, we considered both the unsubstituted propargylamine with the secondary amine (–N(H)–) and its methylated analog (–N(Me)–), based on our earlier work that elucidated the latter to be a significant factor contributing to the higher potency of **SEL** over **RAS [53]**, but also in determining the MAO-B selectivity for *N*-methylhistamine over histamine [55]. This strategy led to 46 different propargylamines whose docking MAO-B binding affinities are given in Table 1. In analyzing the data, we will employ a general notation **XyZ**, such as **2aH** or **4cMe**, where **X** and **y** denote the scaffold number and the position of the attached propargylamine moiety, respectively, while **Z** assumes H or Me given the type of its amino group, –N(H)– or –N(Me)–. On a very general note, we can say that, apart from its crucial warhead role in inactivating MAO-B, the introduced propargylamine group improves the affinity, which represents an interesting insight. A good example is naphthalene **7**, a nonproductive binder with Δ*G*_BIND_ = −6.3 kcal mol^−1^, while all of his four propargylamine derivatives surpass that affinity. This goes even further by transforming **7** into an active site binder **7aH** (Figure 4), linked with the highest potency among all of the considered derivatives, Δ*G*_BIND_ = −7.8 kcal mol^−1^, in line with a range of potent naphthalene-bearing ligands able to inactivate MAO-B [63,64]. Similarly, four-out-of-six diphenylether derivatives have a more exergonic binding than unsubstituted **2**, yet none are preferentially placed within the active site. Still, for **2bH** and **2bMe**, we identified less-favorable active site poses, but with affinity differences exceeding 0.4 kcal mol^−1^, which limits their applicability. In contrast, although unsubstituted iminocoumarin **25** is a more promising lead than coumarin **16**, all of its propargylamine derivatives are nonproductive binders, except **25eH**, which has the third highest active site affinity among all of the systems (Δ*G*_BIND_ = −7.1 kcal mol^−1^). Moreover, **25cH** should also be considered given its high active site affinity, being Δ*G*_BIND_ = −6.9 kcal mol^−1^. On the other hand, coumarin scaffold **16** offered three active site binders, **16bH**, **16cH**, and **16fH**, with an interesting observation that, in all of them, the propargylamine *N*-methylation considerably reduced affinities and even changed the preference to nonproductive positions. Still, this hints at a very preliminary conclusion that, in terms of their size and electronic features, the molecular architecture of coumarin and iminocoumarin scaffolds, when propargylamine-bearing, is somewhere at the borderline for the efficient binding within the bipartite MAO-B substrate cavity. As a result, further *N*-methylation likely crosses that border and predominantly exerts unfavorable effects on the binding profile. Lastly, following the scaffold docking, indole **4** was certainly the least promising skeleton, with its lowest nonproductive affinity Δ*G*_BIND_ = −5.8 kcal mol^−1^, being further reduced to −5.5 kcal mol^−1^ for the active site attachment. Yet, upon derivatization, it offered as much as six active site ligands, with the most potent **4fH** having Δ*G*_BIND_ = −7.5 kcal mol^−1^. This agrees with several literature reports that identified indole as a valuable building block for efficient MAO-B ligands [65,66,67], including potent irreversible inhibitors ASS234 (IC_50_ = 177 nM) [68] and contilisant (IC_50_ = 78 nM) [69].

In ending this section, it is gratifying to see that the propargylamine group had a dominantly positive effect on the binding potency, while ligands **4cH**, **4fH**, **4fMe**, **7aH**, **16cH**, **16fH**, and **25eH** were carried out to the next phase (Figure 4). This choice is justified, seeing that all of these ligands show higher affinity over reference drugs **RAS** (Δ*G*_BIND_ = −5.9 kcal mol^−1^) and **SEL** (Δ*G*_BIND_ = −6.2 kcal mol^−1^). We note in passing that our docking protocol predicted a higher affinity of **SEL** over **RAS**, being in line with their experimental *K*_I_ and IC_50_ values [70], which lends credence to the accuracy of the presented analysis.

### 2.3. Molecular Dynamics Simulations of Selected Propargylamines in the MAO-B Active Site

Seven potent drug candidates that most preferentially bind to the active site, underlined through the docking analysis, were submitted to classical molecular dynamics (MD) simulations to inspect their conformational flexibility within MAO-B and reveal specific protein–ligand interactions governing the binding. The first thing we monitored was the evolution of distances between the terminal C_γ_ atom of the propargylamine unit and the N5 atom of the FAD co-factor (Figure 1), which helped us to evaluate the tendency of each ligand to persist within the active site during 300 ns of MD simulations (Figure S1). It turned out that only the ligands **4fH**, **4fMe**, and **16cH** remained firmly bound within the enzyme, while the other four systems revealed a tendency to depart from it. Specifically, **16fH** and **25eH** almost immediately left the active site, while **7aH** and **4cH** acted the same after 120 and 230 ns (Figure S1), which suggests their very limited inhibition ability. This left us with ligands **4fH**, **4fMe**, and **16cH** for the subsequent binding affinity analysis and its decomposition into contributions from individual residues (Table 2).

It turned out that Δ*G*_BIND_ for all ligands are negative and indicate a favorable binding. This holds for the reference drugs as well, whose binding affinities, Δ*G*_BIND_ = −20.1 kcal mol^−1^ for **SEL** and Δ*G*_BIND_ = −18.1 kcal mol^−1^ for **RAS**, reveal the right trend among values. Still, these are likely overestimated in absolute terms, which is a known limitation of the MM-PBSA approach, as extensively discussed in a recent review by Homeyer and co-workers [71] that also underlined its immense potential in predicting relative binding energies in biomolecular complexes [71], which is the focus here. In this context, the relative difference among ligands of ΔΔ*G*_BIND_ = −2.0 kcal mol^−1^ in favor of **SEL** is found in excellent agreement with −2.6 kcal mol^−1^ predicted from experimental IC_50_ values of 82.5 nM for **RAS** [72] and 1.3 nM for **SEL** [73], which lends confidence to presented conclusions.

**Figure 5:**
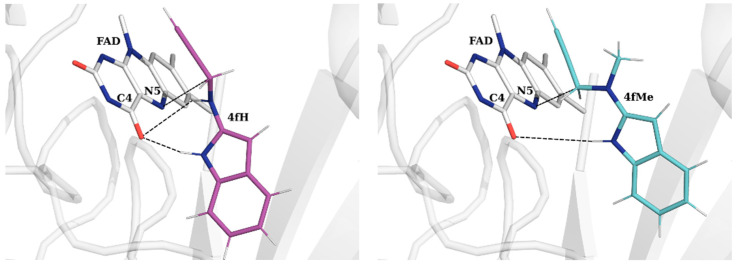
Orientation of **4fH** (left) and **4fMe** (right) towards the FAD co-factor within the MAO-B active site. Dashed lines indicate the relevant distances discussed in the text, including the hydrogen bonding contacts between C4-carbonyl on FAD and both indole (2.11 Å in **4fH**, 1.96 Å in **4fMe**) and propargylamine nitrogens (2.87 Å in **4fH**), as well as reactive coordinates between N5(FAD) and C_α_(ligand) (5.07 Å in **4fH**, 3.21 Å in **4fMe**).

**16cH** appears as the least potent ligand with Δ*G*_BIND_ = −19.7 kcal mol^−1^. Yet, it is largest in size, which is why its contribution from the gating Ile199 at the end of the substrate cavity is highest at −1.45 kcal mol^−1^, corresponding to a series of favorable C–H···π contacts with the aromatic part of the ligand. Given its volume, it provides the most opportunities for interaction with the “aromatic cage” tyrosines, Tyr398, Tyr435, and Tyr326, whose individual contributions, together with that from the FAD co-factor, sum up to the highest value of −10.24 kcal mol^−1^, which account for 52% of its total binding affinity. The high contribution from FAD originates in its interactions with the propargylamine moiety through positive π···π stacking contacts, with the average distance between the reactive N5(FAD) and Cα(**16cH**) being 3.77 Å. This holds for all of the considered five ligands, where it represents the largest individual contribution in all cases. However, the rest of the crucial active site residues exhibit reduced contributions relative to other ligands, thereby resulting in a lower total affinity over reference **SEL**. This is improved in both indoles **4fH** and **4fMe**, whose affinities either match or even surpass that of **SEL** by −1.5 kcal mol^−1^ for the latter. Both ligands significantly advance their contacts with FAD, especially **4fMe**, where the FAD’s contribution is as much as 2 kcal mol^−1^ higher than in **SEL**, being dominantly responsible for its higher affinity. Part of this stems from a very favorable hydrogen bonding that both indole N–H groups are making with FAD C4-carbonyl moiety (Figure 5 and Figure S2). This is stronger in **4fMe**, as seen in its shorter average N–H···O distance of 1.96 Å relative to 2.11 Å in **4fH**. At the same time, the unsubstituted propargylamine N–H group in **4fH** is also making hydrogen bonding contacts with the same FAD C4-carbonyl moiety at d(N–H···O) = 2.87 Å. However, this interaction positions its reactive C_α_ group in a less optimal position for the inactivation reaction (Figure 5), evident in a longer C_α_(ligand)···N5(FAD) distance of 5.07 Å in **4fH** relative to 3.21 Å in **4fMe**. In other words, with its secondary propargylamine nitrogen atom, **4fH** exerts its ability to form two favorable hydrogen bonds with the FAD C4-carbonyl group, which results in a less reactive conformation for the covalent inhibition process. We will further elaborate on this aspect in the last part of the discussion, dealing with the mechanistic calculations.

In contrast, **4fMe** features the tertiary propargylamine nitrogen center that is unable to form any hydrogen bonds with FAD, but rather prefers positioning its N–Me group within the hydrophobic pocket comprising Ile198, Ile199, and Tyr188. This is evident in a consistently higher contribution from these three residues in **4fMe**, where it sums to −1.69 kcal mol^−1^, relative to **4fH**, where it is −1.38 kcal mol^−1^. Such a preference locates the vicinal C_α_–H group in **4fMe** in a position closer to the FAD co-factor, which will determine its larger inactivation potency, as the subsequent discussion will show. This strongly underlines the fact that the ligand capacity to form hydrogen bonds within the active site is not a prerequisite for a successful binding or an efficient covalent inhibition, but other electrostatic and hydrophobic interactions are dominating in this respect. After all, this is precisely the case with **SEL** and **RAS** themselves [53], as much more frequent hydrogen bonding contacts that **RAS** is making with the active site residues only position it into a less reactive conformation over **SEL**. Other than that, we see a high contribution from Gln206 in both indoles that comes as a result of positive side-chain amide N–H···π contacts with the aromatic part of each ligand. We also observe that two “aromatic cage” tyrosines directly hosting the ligand alkyne unit, Tyr398 and Tyr435, show an identical large cumulative effect of −4.38 kcal mol^−1^ in both ligands, which is a noteworthy coincidence that confirms the appropriate placement of this warhead moiety within the MAO-B active site. We note that these two residues were previously identified, both experimentally [74] and computationally [55,75], as important in properly orienting substrates for the chemical reaction, and their mutations have led to enzymes with considerably reduced activities [76]. Analogously, our current study confirms their significance for the inhibitor affinity as well. Lastly, most of the remaining residues promoting the binding of both **4fH** and **4fMe** are hydrophobic in nature (Table 2), which exert their stabilizing effect by being in the vicinity of ligand aromatic parts. With all of this in mind, we decided to submit the derivatives **4fH** and **4fMe** to the mechanistic DFT calculations to further evaluate their inhibition potency through kinetic and thermodynamic parameters underlying the MAO-B inactivation chemical reaction.

### 2.4. DFT Analysis of the Inhibition Reaction

**Figure 6:**
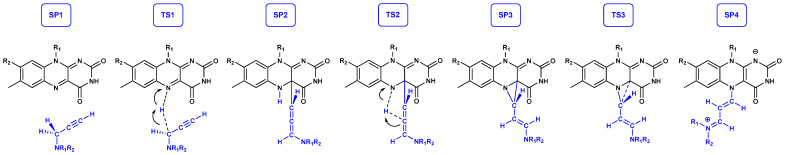
Chemical structures corresponding to stationary points (SP) and transition state structures (TS) during the irreversible MAO inhibition with the rate-limiting hydride transfer reaction during the SP1 → SP2 conversion.

Previously, we used QM cluster calculations [53] and EVB QM/MM simulations [54] to elucidate the precise molecular mechanism behind the irreversible MAO inhibition with the clinical drugs **SEL** and **RAS**. We showed that MAO inactivation occurs through a three-step process (Figure 6), where, in the rate-limiting first step, the enzyme uses the N5(FAD) atom to abstract a hydride anion from the inhibitor C_α_–H group. This was found to be in full analogy with the MAO catalytic mechanism [13], thereby confirming that both **SEL** and **RAS** are mechanism-based inhibitors [2,77]. This process creates a C4a(FAD) adduct and an allene motif on the inhibitor, which proceeds by transferring the N5-hydrogen back to the inhibitor C_β_ atom as a proton, H^+^, thereby forming a three-membered ring with C_γ_ and N5 and C4a atoms on FAD. The last step involves the breaking of the bond between C_γ_(inhibitor)–C4a(FAD) and the formation of a full N5-adduct, thus agreeing with the crystallographic structures (Figure 1). In addition, the computed kinetic and thermodynamic data confirmed the higher potency of **SEL** through a lower activation energy, ΔΔ*G*^‡^ = −1.2 kcal mol^−1^, the latter matching the experimental value of −1.4 kcal mol^−1^ [70], and a more exergonic process, ΔΔ*G*_R_ = −0.8 kcal mol^−1^, both supporting the validity of the proposed hydride mechanism. With this in mind, following MD simulations, we created a cluster model of MAO-B with **4fH**, **4fMe**, and reference drugs **SEL** and **RAS**, including the FAD co-factor and Tyr435, Tyr398, Tyr326, Ile199, Phe343, and Gln206 residues, as described in Section 3. These were submitted to M06–2X/6–31+G(d) geometry optimization and PBE0/6–311++G(d,p) single-point calculations, which gave stationary points corresponding to enzyme–inhibitor complexes.

**Figure 7:**
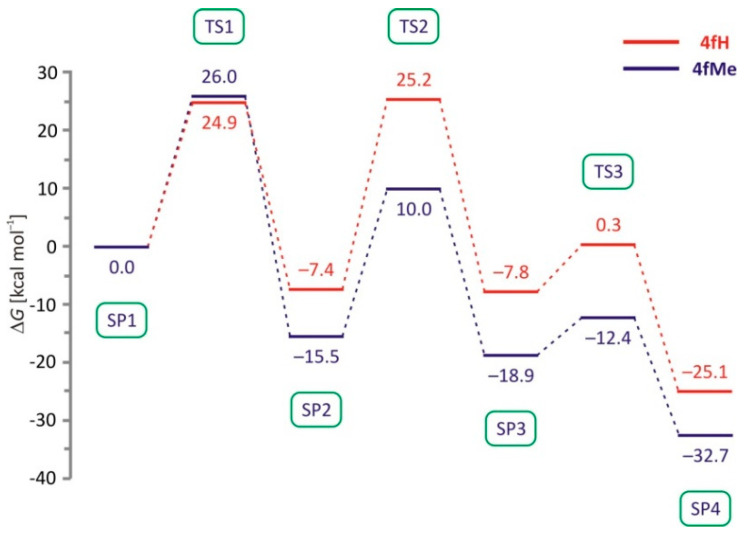
Free-energy profiles for the irreversible MAO-B inhibition with **4fH** and **4fMe**. Acronyms SP and TS indicate stationary points and transition states, respectively, while their general chemical structures are depicted in Figure 6. The rate-limiting step for **4fMe** is the SP1 → SP2 transition with ∆G^‡^ = 26.0 kcal mol^−1^, while for **4fH** it is the SP2 → SP3 conversion with ∆G^‡^ = 32.6 kcal mol^−1^.

Direct C_α_–H hydride abstraction turned out as feasible in both inhibitors (Figure 7). In the transition state TS1, the transferring hydrogen is placed between the leaving α–carbon and the accepting N5 atom, with bond distances of between 1.43 and 1.26 Å for **4fH**, respectively, matching those in **4fMe** at between 1.48 and 1.26 Å, in the same order. Despite these similarities, the activation-free energy for this process in **4fH** is 24.9 kcal mol^−1^, which is increased to 26.0 kcal mol^−1^ in **4fMe**. If this was the rate-limiting step for both ligands, it would predict **4fH** to be around six times a more efficient inhibitor. Yet, following the hydride abstraction, the **4fH** carbocation is stabilized to an allene adduct at −7.4 kcal mol^−1^, while in **4fMe**, this is noticeably more exergonic at −15.5 kcal mol^−1^. In both cases, the adduct features a strong C_γ_(inhibitor)–C4a(FAD) covalent bond of between 1.55 Å for **4fH** and 1.56 Å for **4fMe**. The allene nature of SP2 is evidenced in an even distribution of C_α_–C_β_ and C_β_–C_γ_ bonds of between 1.31 and 1.30 Å in **4fH**, and between 1.32 and 1.31 Å in **4fMe**, respectively. From that point, the succeeding reaction cleaves the formed N5(FAD)–H bond and transfers the hydrogen as a proton H^+^ onto the most nucleophilic allene C_β_ atom, which is highly demanding in **4fH**, going over the highest point on the free energy surface. The latter represents the rate limiting step of the entire conversion with **4fH**, with the activation barrier of Δ*G*^‡^ = 32.6 kcal mol^−1^ being much more challenging than in **4fMe**. In contrast, **4fMe** achieves the SP2 → SP3 reaction over a significantly lower barrier of 25.5 kcal mol^−1^ and forms a much more exergonic intermediate SP3. In line with our earlier results, during the SP2 → SP3 process, an analogous proton transfer to C_γ_ is not feasible, while that of C_α_ reverts the system to SP1 and is not productive. The last step is highly exergonic and requires almost identical activation free energies in both ligands to offer a very stable adduct SP4 as the final product, which features a fully formed N5(FAD)–C_γ_ covalent bond of between 1.32 Å in **4fH** and 1.35 Å in **4fMe**. The rest of the adduct represents a conjugated cyanine structure that accommodates the excess positive charge, with C_γ_–C_β_, C_β_–C_α_ and C_α_–N distances of 1.41, 1.37, and 1.35 Å in **4fH**, while being 1.38, 1.40, and 1.32 Å in **4fMe**, respectively. Overall, **4fMe** proved to be a more effective inhibitor than **4fH** with a lower activation energy, Δ*G*^‡^ = 26.0 kcal mol^−1^, and a more favorable reaction exergonicity, Δ*G*_R_ = −32.7 kcal mol^−1^.

To demonstrate the validity of the hydride transfer mechanism, we utilized an NBO population analysis focusing on atomic charges on relevant sites during the inactivation reaction with a more potent **4fMe** ligand. In the reactive complex (SP1), the charge on the carbon atom undergoing the H^−^ abstraction (C_α_) is −0.34 |e|, while on the accepting N5(FAD) it is −0.35 |e|. At the same time, the total charge on the entire **4fMe** is 0.00 |e|, while on FAD it is 0.02 |e|, which indicates that no charge is exchanged among the reactants before the reaction. In the transition state TS1, the charge on C_α_ becomes more positive at −0.21 |e|, while that on N5(FAD) gains a more negative value of −0.51 |e|. Jointly, this confirms the anionic nature of the transferring hydrogen. We note that the C_α_ atom undergoing the H^−^ abstraction experiences a somewhat smaller change in the atomic charge among the interacting partners, which is rationalized by the presence of the neighboring amino group that compensates for the charge loss through electron donation. This is seen in the reduced charge on the latter and a shortened N(amino)–C_α_ distance in **4fMe**, changing from −0.47 |e| and 1.45 Å in SP1 to −0.43 |e| and 1.42 Å in TS1, all being consistent with the proposed H^−^ transfer. This is further supported by the 0.40 |e| total charge on **4fMe** and −0.39 |e| on FAD in TS1, which indicate that around 40% of the electron charge density is transferred along with the hydrogen atom during the rate-limiting first step. In addition, the atomic charge on N1(FAD) changes from −0.36 |e| in SP1 to −0.39 |e| in TS1, thereby indicating that some of the relocated anionic charge resides on that site through resonance, to assume −0.44 |e| in the final adduct SP4 (Figure 1). The latter agrees with very recent mass spectrometry data that reveals the deprotonated nature of the adduct N1 site even under highly acidic conditions (pH ≈ 2) [78], which all confirms our mechanistic proposal.

Lastly, to evaluate the quality of these results, we have utilized the same approach on the reference drugs **RAS** and **SEL**, whose reaction profiles are presented in Figure S3. Our results confirm a higher efficiency of **SEL** through a lower barrier ΔΔ*G*^‡^ = −1.3 kcal mol^−1^, being fully in line with the determined *k*_inact_ values of 0.53 min^−1^ for **SEL** and 0.05 min^−1^ for **RAS** [70]. In addition, these experimental parameters translate to a difference in the activation free-energy of −1.4 kcal mol^−1^ in favor of **SEL**, which puts our computed value in an almost perfect agreement with experiments and lends strong credence to our results. More importantly, all of this indicates that the kinetic requirements calculated for **4fMe** (Δ*G*^‡^ = 26.0 kcal mol^−1^) suggest it to be around 69 times more potent inhibitor than **SEL** (Δ*G*^‡^ = 28.5 kcal mol^−1^), which highlights **4fMe** as a very promising lead compound whose synthesis and biological evaluation are very much advised.

## 3. Materials and Methods

### 3.1. System Preparation

The starting point for our computations was the high-resolution (1.7 Å) X-ray structure of MAO-B in a complex with *N*-methyl-1-aminoindan [79] obtained from the Protein Data Bank (accession code 2C67). Original crystal waters were removed from the structure for the docking analysis, but also to assure that, during the subsequent molecular dynamics simulations, the water molecules from the bulk solvent could diffuse into the active site during equilibration and production runs. The protein exists as a homodimer with a covalently bound FAD co-factor to the conserved Cys397 residue in each subunit. Protonation states of ionizable residues were set according to PROPKA3.11 server predictions [80] and by inspecting hydrogen bonding networks in their closest vicinity, while the missing hydrogen atoms were added using the *tleap* module in AmberTools16 [81].

### 3.2. Docking Simulations and Cross-Docking Validation

Following the mentioned system preparation, we performed docking simulations of the selected 25 organic scaffolds and, subsequently, 46 propargylamine ligands using the AutoDock Vina algorithm [82] through the UCSF Chimera 1.16. program [83], which operates based on dispersion, hydrogen bonds, electrostatic, and desolvation components to determine the most probable complex conformation. The considered ligands were prepared for the docking simulations with the DockPrep tool within the UCSF Chimera 1.16. software [83], while their protonation states were verified with the MarvinSketch program at pH = 7.2. In this context, all of the ligands were considered to be neutral unionized systems, in line with typical p*K*a values for the present functional groups. The preparation of the grid map was performed using AutoDock Vina, with a grid size set to 60 × 60 × 60 points. During docking simulations, both the MAO-B enzyme and the ligands were treated as rigid. The ten most exergonic ligand positions were manually analyzed and visualized, and favorable poses of a selected number of promising ligands were submitted to the molecular dynamics simulations. In addition, since docking simulations held an important role in discriminating between productively and non-productively bound ligands, we performed a cross-docking validation of the utilized docking procedure [84,85,86] using the AutoDock Vina and AutoDock 4 programs [82], which further underlined its accuracy (for details see Supplementary Materials).

### 3.3. Molecular Dynamics Simulations

For the FAD co-factor and considered ligands, geometry optimization and RESP charge calculations were performed with the Gaussian 16 program [87] at B3LYP/6–31G(d) and HF/6–31G(d) levels, respectively, while the enzyme was modeled using the AMBER ff14SB force field. The initial position of each ligand was taken from the preceding docking simulations, and thus the formed complexes were solvated in a truncated octahedral box of TIP3P waters spanning 10 Å thick buffers. The system was neutralized with 5 Na^+^ ions that were distributed around the system using the Monte-Carlo method within the CHARMM-GUI server, maintaining the net zero charge to simulate periodic boundary conditions. The prepared simulation boxes were submitted to geometry optimization in the GROMACS 2020.4 program [88]. Optimized systems were gradually heated from 0 to 300 K and equilibrated during 250 ps using NVT conditions, followed by productive and unconstrained MD simulations of 300 ns employing a time step of 2 fs at a constant pressure (1 atm) and temperature (310 K). The long-range electrostatic interactions were calculated using the Particle Mesh Ewald method [89], and were updated in every second step, while the non-bonded interactions were truncated at 10.0 Å.

### 3.4. Binding Free Energy Calculations

The binding free energies, Δ*G*_BIND_, for each ligand for MAO-B were calculated by the established MM-PBSA protocol using the g_mmpbsa utility available in the GROMACS 2020.4 program [90]. For that purpose, every tenth structure from the corresponding MD trajectories was utilized, resulting in a total of 30.000 considered snapshots. The calculated MM-PBSA binding free energies were decomposed into specific residue contributions on a per-residue basis according to the established procedure. This protocol calculates contributions to Δ*G*_BIND_ arising from each amino acid residue, and identifies the nature of the energy change in terms of its interaction and solvation energies or entropic contributions.

### 3.5. DFT Mechanistic Analysis

Following MD simulations, we selected a representative structure for each inhibitor, assuring that all of the ligands were found to be in a reactive orientation with their propargylamine groups pointing towards FAD. This allowed us to build a cluster model of the MAO-B enzyme by extracting the initial positions of each inhibitor, FAD co-factor, and Ile199, Gln206, Tyr326, Phe343, Tyr398, and Tyr435 residues. This selection was determined through the preceding MM-PBSA analysis that identified them as important for the ligand binding, but also prompted by the analogous cluster recently employed by Fierro and co-workers [91] in elucidating differences in the catalytic activity of MAO-B towards *p*-chloro- and *p*-methoxy-β-methylphenethylamine. The FAD co-factor was truncated at the ethyl group on its N10 atom, while all of the selected amino acids were truncated at their α-carbons, which were kept in the form of the methyl group. To minimize errors associated with the initial selection of starting geometries from MD trajectories, we tried several conformations of each substrate within the so-formed cluster and proceeded with the mechanistic calculations using the most stable complexes.

As a good compromise between accuracy and computational feasibility, all of the geometries were optimized by the very efficient M06–2X/6–31+G(d) method with thermal Gibbs free energy corrections extracted from the corresponding frequency calculations without the scaling factors. The final single-point energies were refined with a highly flexible 6–311++G(d,p) basis set employing the PBE0 DFT functional, in line with our previous work, where an analogous approach was utilized to elucidate the catalytic [13] and inhibition [53] mechanisms, as well as the selectivity [55] of MAO-B enzymes, but was also successfully applied to other biological systems as well [92,93]. In the utilized cluster approach, a truncated but carefully selected part of the enzyme was treated with the quantum mechanical methodology. To account for the long-range electrostatic interactions and polarization effects caused by the rest of the enzyme, we included a conductor-like polarisable continuum model (CPCM), with a common dielectric constant of ε = 4 and other parameters corresponding to pure water. This approximation assumes that the enzyme’s surrounding is a homogeneous polarizable medium characterized by a dielectric constant ε. This yields the (CPCM)/PBE0/6–311++G(d,p)//(CPCM)/M06–2X/6–31+G(d) model used here. In this approach, the steric influence that the enzyme matrix imposes on the active site is usually modeled by fixing the position of certain atoms, typically where the truncation of the residues is made. Yet, this could, especially for smaller models, lead to a too-rigid cluster having an artificial strain, which can result in wrong energy profiles. Larger models, such as the one employed here, usually grant enough flexibility to accommodate changes that take place during the reaction, and these models have been shown to generally yield good results [94,95]. For those reasons, we did not fix the position of any atom during the geometry optimization and obtained stable stationary points and the resulting free-energy profiles. The validity of the employed computational approach is further prompted by its success in offering relative kinetic and thermodynamic results for reference drugs rasagiline and selegiline in excellent quantitative agreement with available experiments. All of the transition state structures were verified to have the appropriate imaginary frequencies, from which the corresponding reactants and products were determined by the intrinsic reaction coordinate (IRC) procedure. Atomic charges were obtained by natural bond orbital (NBO) analyses [96] as the single-point calculations at the (CPCM)/M06–2X/6–31+G(d) level. All of the calculations were performed using the Gaussian 16 software [88].

## 4. Conclusions

Our computational strategy in designing potent and efficient MAO-B inhibitors relied on considering 20 different organic scaffolds identified in the ChEMBL20 database to have the highest cumulative counts of unique member compounds and bioactivity assays [49], together with five additional skeletons based on our experience in their derivatization towards a range of photophysical and biological activities. These compounds were docked to the MAO-B enzyme, revealing coumarin (**16**) and iminocoumarin (**25**) to be the preferable active-site binders. Additionally, diphenylether (**2**), indole (**4**), and naphthalene (**7**) were found to exhibit placements within MAO-B active sites that are only between 0.1 and 0.3 kcal mol^−1^ less exergonic over their most favorable nonproductive poses. The five highlighted scaffolds were fully mono-substituted with the propargylamine core as a common functional group responsible for the irreversible MAO-B inactivation through the covalent adduct formation with its FAD co-factor, while the fine-tuning of the proposed ligands was attempted by exchanging the propargylamine N–H group with the N–Me moiety. This led to 46 different propargylamines that were submitted to docking simulations, with a consistent conclusion that the introduced propargylamine unit generally increased the affinity, while even changing the preference from nonproductive to active site binding in indole derivatives. As a result, seven different ligands with indole, naphthalene, coumarin, and iminocoumarin scaffolds showed higher active site affinities over the clinical drugs **RAS** and **SEL**. These were further analyzed through the classical molecular dynamics simulations to inspect their conformational flexibility within the MAO-B enzyme and revealed specific protein–ligand interactions governing the binding. The results show that only ligands **4fH**, **4fMe**, and **16cH** remained firmly bound within the enzyme, while the other four derivatives demonstrated a tendency to depart it during 300 ns of MD simulations. Out of the three ligands with a favorable binding profile, only indole-2-propargylamine **4fH** and indole-2-*N*-methylpropargylamine **4fMe** exerted affinities similar or surpassing that for **SEL**, respectively. Specifically, the binding of **4fMe** turned out as more exergonic by −1.6 kcal mol^−1^, being predominantly afforded by a more favorable placement of its propargylamine unit towards both FAD and “aromatic-cage” tyrosines, and the positioning of its N–Me unit within the hydrophobic pocket involving Ile198, Ile199, and Tyr188. The latter is seen in a consistently higher joint contribution from these three residues in **4fMe** (−1.69 kcal mol^−1^), relative to that in **4fH** (−1.38 kcal mol^−1^). In contrast, the unsubstituted propargylamine N–H group in **4fH** displayed its ability to form additional hydrogen bonding with the C4-carbonyl group on FAD, which only positions this ligand in a less reactive conformation for the inactivation reaction.

The most promising derivatives **4fH** and **4fMe** were submitted to the mechanistic DFT calculations within the MAO-B cluster model, which showed that both inhibitors followed the three-step hydride mechanisms [53], where, in the first step, the ligand undergoes the hydride abstraction from its C_α_–H group onto the FAD N5 atom, thus creating a C4a(FAD) adduct and an allene motif on the inhibitor. This is followed by the proton transfer from the newly formed N5–H group to the inhibitor C_β_ atom. The latter creates a strained three-membered ring with C_γ_, N5, and C4a atoms on FAD, which transform into the fully formed N5-adduct, thereby tying in with crystallographic data collected on inactivated enzymes. Although this mechanistic scenario is feasible with both of the considered ligands, the obtained reaction profiles point to a much higher efficiency of **4fMe**, seen in a lower activation free energy, Δ*G*^‡^ = 26.0 kcal mol^−1^, and a more favorable reaction exergonicity, Δ*G*_R_ = −32.7 kcal mol^−1^. Together with a more favorable binding affinity over clinically employed **SEL**, consistently determined through both docking (ΔΔ*G*_BIND_ = −0.1 kcal mol^−1^) and MM-PBSA MD simulations (ΔΔ*G*_BIND_ = −1.5 kcal mol^−1^), the latter indicates its faster MAO-B inactivation through reduced kinetic requirements (ΔΔ*G*^‡^ = −2.5 kcal mol^−1^) and an improved reaction exergonicity (ΔΔ*G*_R_ = −4.3 kcal mol^−1^) over **SEL**, thereby promoting indole-2-*N*-methylpropargylamine **4fMe** to an excellent drug candidate whose synthesis and biological evaluation are highly recommended.

Lastly, to verify the accuracy of our results, the relative data among the clinical drugs selegiline (**SEL**) and rasagiline (**RAS**) were found to be in excellent agreement with experiments. Specifically, both the docking and MM-PBSA MD simulations pointed to a higher affinity of **SEL** (ΔΔ*G*_BIND_ = −2.0 kcal mol^−1^), thus matching the value of −2.6 kcal mol^−1^ suggested from determined IC_50_ values, while the computed difference in kinetic requirements for the inhibition, ΔΔ*G*_BIND_ = −1.3 kcal mol^−1^ in favor of **SEL**, agrees with −1.4 kcal mol^−1^ advised by the available *k*_inact_ values for both inhibitors. We believe all of this lends credibility to the results presented in this study and the conclusions derived thereof.

## Figures and Tables

**Table 1:** Binding affinities and modes of binding for organic scaffolds substituted with the propargylamine group at designated positions as obtained with the docking simulations (in kcal mol^−1^).

Scaffold	Position	–N(X)– ^a^	Binding	Mode of Binding	Active Site Binding
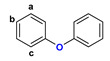 **2**	a	N–H	−6.8	nonproductive	–
		N–Me	−6.9	nonproductive	–
	b	N–H	−7.0	nonproductive	−6.6
		N–Me	−7.4	nonproductive	−6.9
	c	N–H	−6.4	nonproductive	–
		N–Me	−5.9	nonproductive	–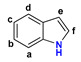 **4**	a	N–H	−5.9	nonproductive	–
		N–Me	−6.4	active site	
	b	N–H	−6.2	nonproductive	−6.2
		N–Me	−5.9	nonproductive	–
	c	N–H	−6.8	active site	
		N–Me	−6.6	active site	
	d	N–H	−6.5	active site	
		N–Me	−6.0	nonproductive	–
	e	N–H	−5.7	nonproductive	–
		N–Me	−5.9	nonproductive	–
	f	N–H	−7.5	active site	
		N–Me	−6.3	active site	
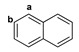 **7**	a	N–H	−7.8	active site	
		N–Me	−6.4	nonproductive	–
	b	N–H	−6.4	nonproductive	−6.3
		N–Me	−6.4	nonproductive	–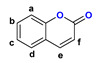 **16**	a	N–H	−6.0	nonproductive	–
		N–Me	−6.3	nonproductive	–
	b	N–H	−6.2	active site	–
		N–Me	−6.1	nonproductive	–
	c	N–H	−6.9	active site	–
		N–Me	−5.7	nonproductive	–
	d	N–H	−6.3	nonproductive	–
		N–Me	−6.0	nonproductive	–
	e	N–H	−6.6	active site	–
		N–Me	−6.1	nonproductive	–
	f	N–H	−6.2	nonproductive	–
		N–Me	−5.8	nonproductive	–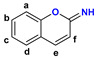 **25**	a	N–H	−6.6	nonproductive	–
		N–Me	−6.2	nonproductive	–
	b	N–H	−6.3	nonproductive	–
		N–Me	−6.8	nonproductive	–
	c	N–H	−7.1	nonproductive	−6.9
		N–Me	−6.5	nonproductive	–
	d	N–H	−6.7	nonproductive	–
		N–Me	−6.1	nonproductive	–
	e	N–H	−7.1	active site	
		N–Me	−6.2	nonproductive	−5.4
	f	N–H	−6.2	nonproductive	–
		N–Me	−6.3	nonproductive	–
Rasagiline **RAS**			−5.9	active site	
Selegiline **SEL**			−6.2	active site	

^a^ Denotes the propargylamine amino group type, either secondary (–N(H)–) or tertiary (–N(Me)–).

**Table 2:** Binding free energies (Δ*G*_BIND_) from MD trajectories using the MM-PBSA approach, and their decomposition on a per-residue basis ^a^.

Ligand	SEL	RAS	4fH	4fMe	16cH
**Δ*G*_BIND_**	−20.1	−18.1	−20.0	−21.6	−19.7
FAD	−2.87	−3.00	−4.63	−4.84	−3.13
Tyr398	−2.43	−1.70	−2.07	−2.13	−2.44
Tyr435	−2.26	−2.43	−2.31	−2.25	−2.15
Gln206	−1.81	−1.62	−2.21	−2.56	−1.37
Leu171	−1.15	−1.81	−0.96	−0.89	−0.96
Cys172	−1.15	−0.99	−0.59	−0.76	−0.83
Ile199	−1.00	−0.57	−0.98	−1.06	−1.45
Phe343	−1.00	−0.80	−0.98	−1.19	−0.28
Tyr326	−0.80	−0.90	−0.74	−0.69	−2.52
Ile198	−0.63	−0.75	−0.18	−0.34	−0.29
Tyr60	−0.56	−0.19	−0.75	−1.01	−0.27
Tyr188	−0.51	−0.40	−0.22	−0.29	−0.19
Gly434	−0.48	−0.35	−0.44	−0.32	−0.41
Leu328	−0.32	−0.17	−0.37	−0.39	−0.43
Trp432	−0.20	−0.13	−0.16	−0.15	−0.14
Val173	−0.19	−0.11	−0.14	−0.18	−0.10
Thr399	−0.19	−0.09	−0.14	−0.10	−0.06
Cys192	−0.18	−0.08	−0.05	−0.06	−0.08
Gly205	−0.14	−0.10	−0.25	−0.21	−0.13
Met341	−0.13	−0.07	−0.10	−0.30	−0.06
Phe168	−0.11	−0.31	−0.07	−0.09	−0.23

^a^ Residues are selected to list all of those with contributions higher than −0.10 kcal mol^−1^ for the reference drug selegiline (**SEL**), as these account for around 90% of its total binding affinity. Residues disfavoring the binding have much smaller contributions not exceeding +0.20 kcal mol^−1^ in all of the studied ligands, and thus are not listed. All values are in kcal mol^−1^.

## Data Availability

The original contributions presented in this study are included in the article/Supplementary Materials; further inquiries can be directed to the corresponding author. All input files required to reproduce all of the reported results are available at https://github.com/lvrban2/Monoamine-oxidase-B-and-indole-2-N-methylpropargylamine/tree/main.

## Supplementary Materials

The following supporting information can be downloaded at: https://www.mdpi.com/article/10.3390/ph17101292/s1, Figure S1. Evolution of the distances between the N5 atom on the FAD co-factor and the terminal C(γ) atom on selected propargylamine inhibitors during 300 ns of MD simulations. Figure S2. Evolution of selected distances for systems **4fH** and **4fMe** bound to the MAO-B active site during 300 ns of MD simulations. Figure S3. Free-energy profiles for the irreversible MAO-B inhibition with **RAS** and **SEL**. Details of the cross-docking validation.
