# Supplementary material for: Prominent Neuroprotective Potential of Indole-2-N-methylpropargylamine: High Affinity and Irreversible Inhibition Efficiency towards Monoamine Oxidase B Revealed by Computational Scaffold Analysis
Source: Pharmaceuticals (Basel). 2024 Sep 28;17(10):1292. doi: 10.3390/ph17101292 (PMC11510145; doi:10.3390/ph17101292)
Supplement: Supplementary file 1 [file pharmaceuticals-17-01292-s001.zip › pharmaceuticals-3196094-supplementary.pdf]

SUPPORTING INFORMATION

Prominent neuroprotective potential of indole-2-N-methylpropargylamine: High affinity and irreversible inhibition efficiency towards monoamine oxidase B revealed by computational scaffold analysis

Lucija Vrban and Robert Vianello*

Laboratory for the Computational Design and Synthesis of Functional Materials, Division of Organic Chemistry and Biochemistry, Ruđer Bošković Institute, Zagreb, Croatia.

*Corresponding author. Email: robert.vianello@irb.hr

TABLE OF CONTENTS

Content	Page
**Figure S1.** Evolution of the distances between the N5 atom on the FAD co-factor and the terminal C(γ) atom on selected propargylamine inhibitors during 300 ns of MD simulations. Data indicate that only systems **4fH**, **4fMe** and **16cH** remain within the MAO-B active site throughout the entire simulation time.	S2
**Figure S2.** Evolution of selected distances for systems **4fH** and **4fMe** bound to the MAO-B active site during 300 ns of MD simulations.	S3
**Figure S3.** Free-energy profiles for the irreversible MAO-B inhibition with **RAS** (in red) and **SEL** (in blue). Acronyms SP and TS indicate stationary points and transition states, respectively, while their general chemical structures are depicted below.	S4
Details of the cross-docking validation	S5–S6
References	S7

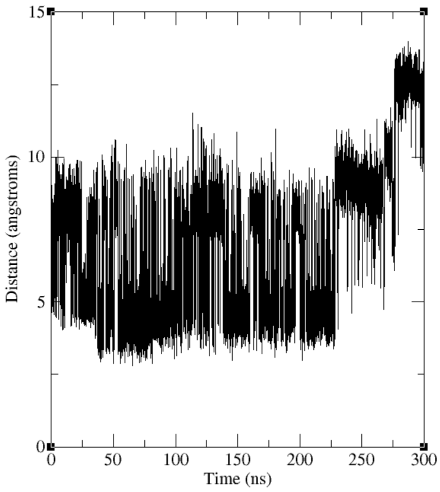

4cH

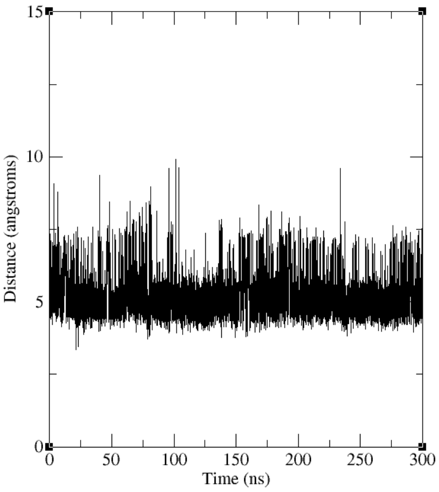

4fH

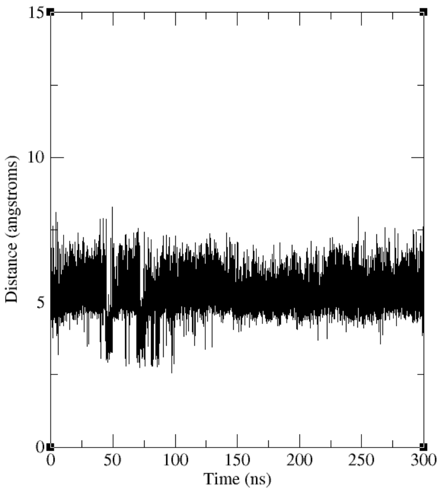

4fMe

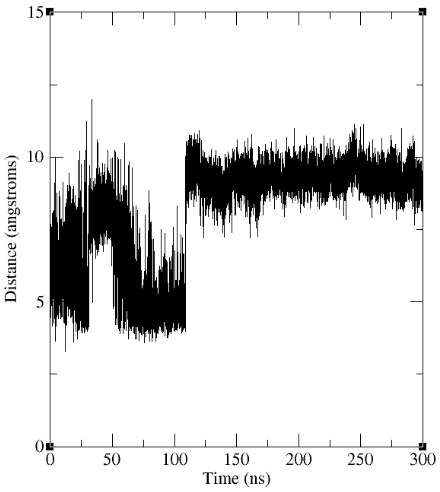

7aH

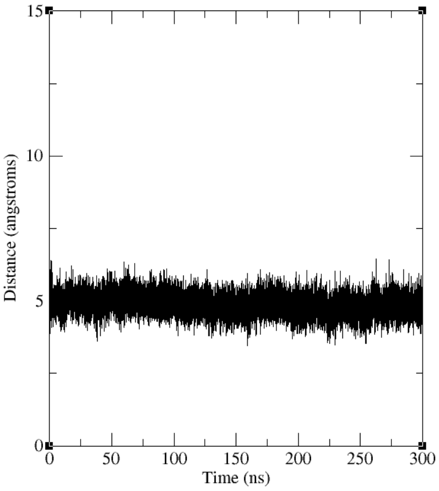

16cH

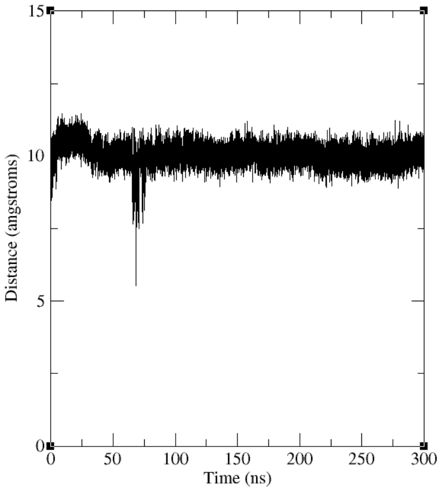

16eH

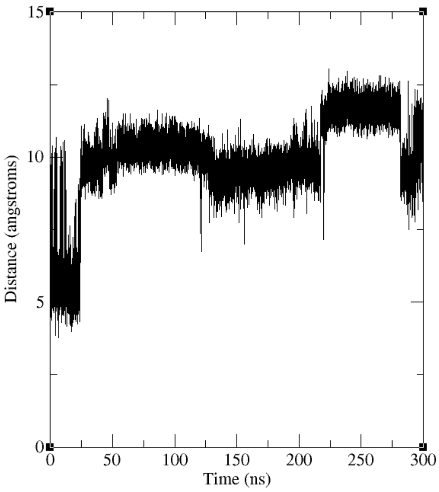

25eH

Figure S1. Evolution of the distances between the N5 atom on the FAD co-factor and the terminal C(γ) atom on selected propargylamine inhibitors during 300 ns of MD simulations. Data indicate that only systems **4fH**, **4fMe** and **16cH** remain within the MAO-B active site throughout the entire simulation time.

Ligand	Cα(ligand)···N5(FAD)	N–H(indole)···O=C4(FAD)	N–H(propargylamine)···O=C4(FAD)
**4fH**	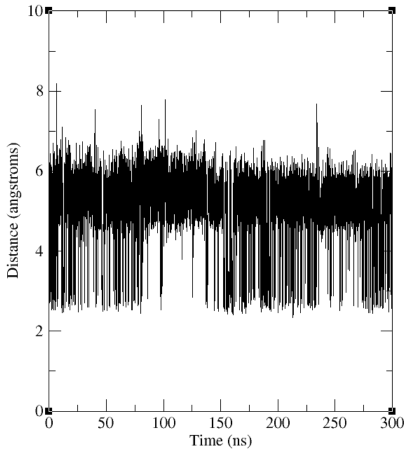	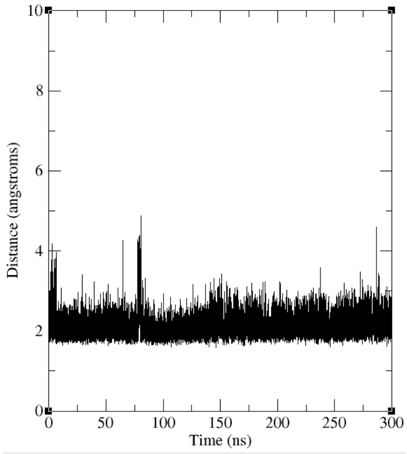	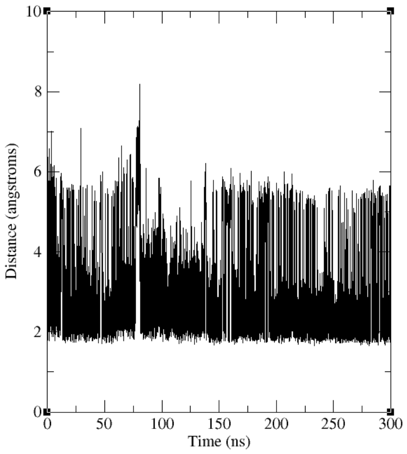
	Average distance = 5.07 Å	***Average distance = 2.11 Å***	***Average distance = 2.87 Å***
**4fMe**	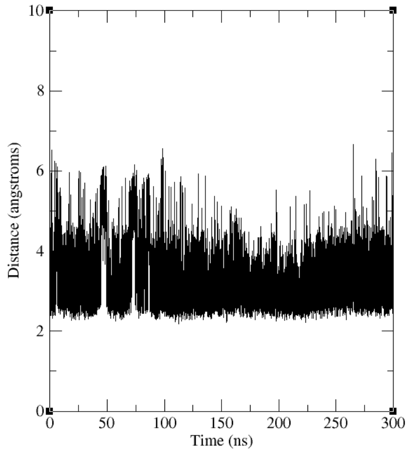	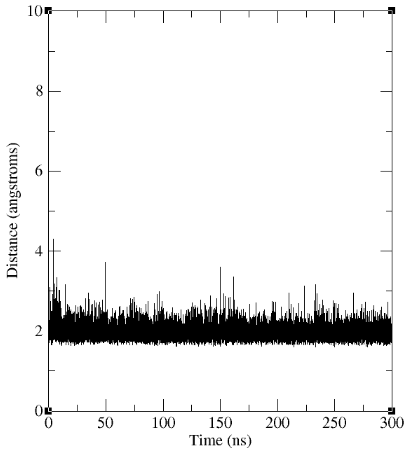	
	Average distance = 3.21 Å	***Average distance = 1.96 Å***	

Figure S2. Evolution of selected distances for systems **4fH** and **4fMe** bound to the MAO-B active site during 300 ns of MD simulations.

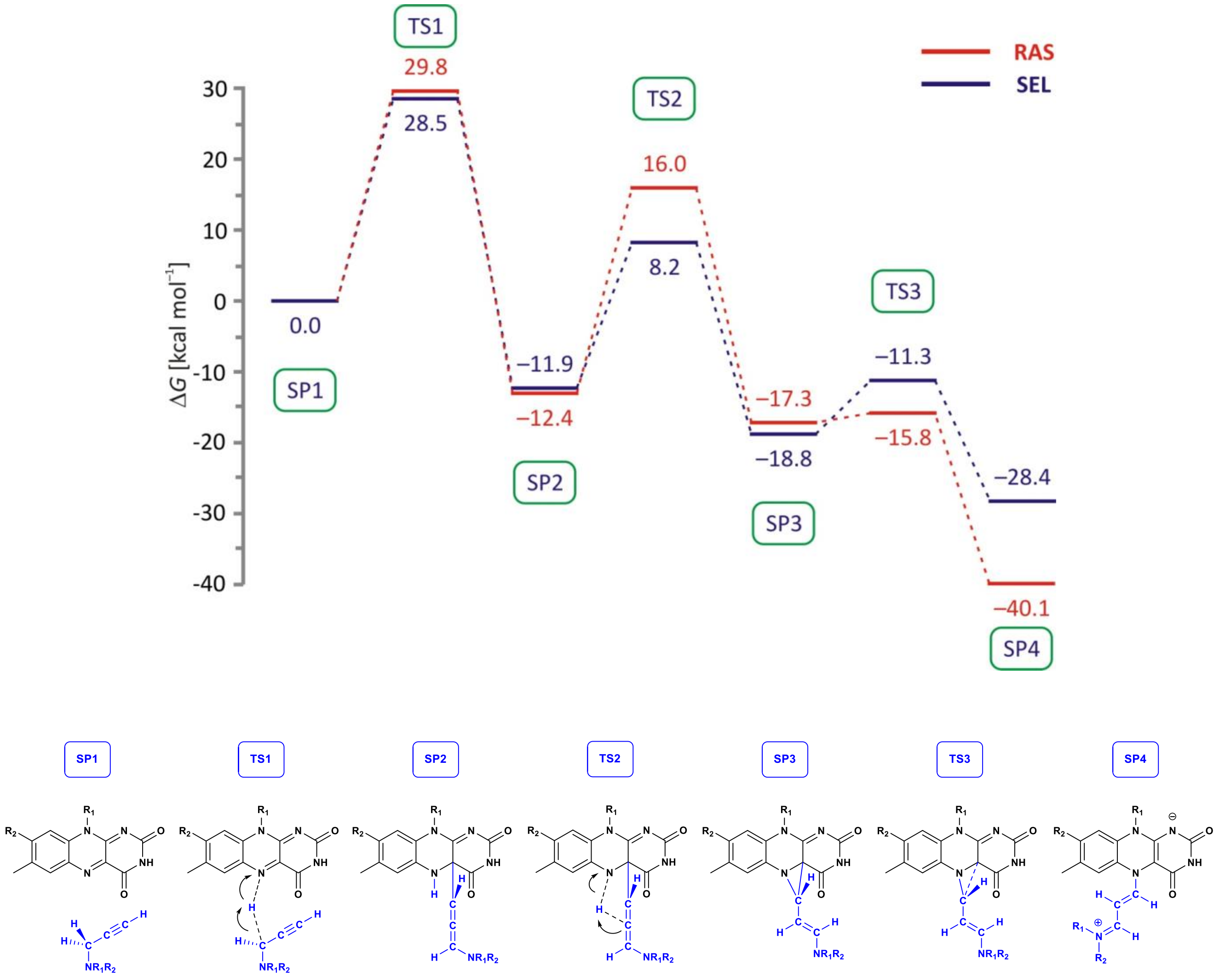

Figure S3. Free-energy profiles for the irreversible MAO-B inhibition with **RAS** (in red) and **SEL** (in blue). Acronyms SP and TS indicate stationary points and transition states, respectively, while their general chemical structures are depicted below.

DETAILS OF THE CROSS-DOCKING VALIDATION

Since docking simulations held an important role in discriminating between productively and non-productively bound ligands and provided starting geometries for the subsequent MD simulations, we found it necessary to perform a cross-docking validation of the utilized docking procedure [1–3] using the AutoDock Vina and AutoDock 4 programs. Because of a large number of the resolved crystallographic MAO-B structures with different conformations, the suitability of the selected 2C67.pdb structure, probed through its ability to accommodate a chemically diverse set of ligands with acceptable RMSD values, was investigated against 4 other structures with the following parameters:

PDB Code	Crystallized Ligand	Resolution	Reference
2C67	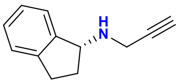	1.70 Å	C. Binda, F. Hubalek, M. Li, Y. Herzig, J. Sterling, D.E. Edmondson, A. Mattevi, J Med Chem 48 (2005) 8148.
2V61	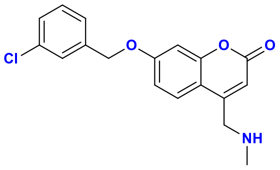	1.70 Å	C. Binda, J. Wang, L. Pisani, C. Caccia, A. Carotti, P. Salvati, D.E. Edmondson, A. Mattevi, J Med Chem 50 (2007) 5848.
3PO7	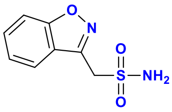	1.80 Å	C. Binda, M. Aldeco, A. Mattevi, D.E. Edmondson, J Med Chem 54 (2011) 909–912.
4A79	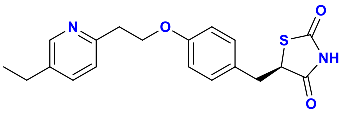	1.89 Å	C. Binda, M. Aldeco, W.J. Geldenhuys, M. Tortorici, A. Mattevi, D.E. Edmondson, ACS Med Chem Lett 3 (2012) 39–42.
6FWC	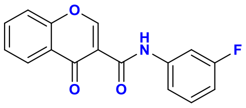	1.70 Å	J. Reis, N. Manzella, F. Cagide, J. Mialet-Perez, E. Uriarte, A. Parini, F. Borges, C. Binda, J Med Chem 61 (2018) 4203–4212.

Initially, we performed a re-docking protocol, which tests docking parameters and evaluates its capacity to reproduce both the co-crystallized binding geometry and the associated ligand orientation given a rigid macromolecule state. During this, the docked pose of each ligand is gauged against its crystal structure orientation within the same protein. The quality of the method is assessed through the RMSD data, where RMSD parameters less than 2 Å are considered as satisfactory [1].

PDB Code	Crystallized Ligand	Re-Docking RMDS
2C67	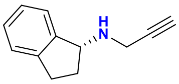	0.746
2V61	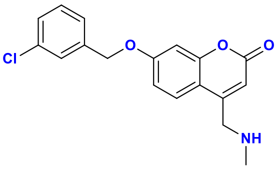	3.052
3PO7	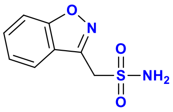	2.598

4A79	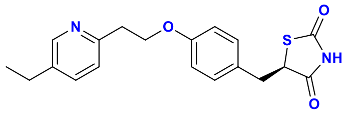	0.960
6FWC	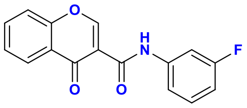	0.548

The obtained data revealed that three structures qualify as appropriate (2C67, 4A79, 6FWC) with RMSD parameters below 2 Å, noting that the employed 2C67 structure ranks as second best according to that criterion.

Next, we proceeded with the cross-docking analysis, which inspects how well each protein structure can accommodate non-native ligands. For that purpose, we utilized only structures 2C67, 4A79 and 6FWC, which were identified through re-docking as superior, and all five considered ligands to test against all three single rigid protein structures. In this case, each crystal structure provides a reference for where the ligand should dock if the binding cavity of the probed protein structure can accommodate it. Here, the success of the method is again evaluated relative to the RMSD data between the docked pose and the respective reference conformation. The cross-docking protocol offered the following RMSD parameters:

**Crystal Structure**		**Cross-Docked Structure**		
**PDB Code**	**Ligand**	**2C67**	**4A79**	**6FWC**
2C67	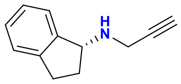	–	No Active Site Binding	6.494
2V61	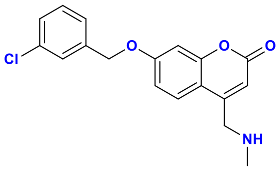	1.397	No Active Site Binding	1.957
3PO7	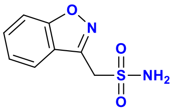	5.368	2.141	5.028
4A79	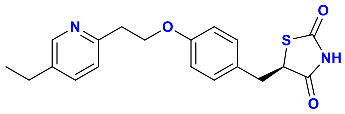	0.981	–	No Active Site Binding
6FWC	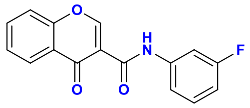	0.813	No Active Site Binding	–

It turned out that only 2C67 was able to successfully dock all four ligands within the binding site, with only the zonisamide ligand from the 3PO7 crystal structure exceeding the acceptable RMSD value of 2 Å. The other two considered protein structures performed much worse, seen in their significantly higher RMDS parameters and occasional inabilities to accommodate inspected ligands within the binding site at all. This insight, together with the fact that the 2C67 structure was the second most successful according to the re-docking analysis, highlights it as the most appropriate for screening multiple diverse compounds and confirms its selection for the computational analysis presented in our work.

REFERENCES

[1] Liu K, Kokubo H (2017) Exploring the Stability of Ligand Binding Modes to Proteins by Molecular Dynamics Simulations: A Cross-docking Study. J Chem Inf Model 57: 2514–2522. https://doi.org/10.1021/acs.jcim.7b00412

[2] Liu K, Kokubo H (2020) Prediction of ligand binding mode mong multiple cross-docking poses by molecular dynamics simulations. J Comput Aided Mol Des 34: 1195–1205. https://doi.org/10.1007/s10822-020-00340-y

[3] Mateev E, Valkova I, Angelov B, Georgieva M, Zlatkov A (2022) Validation through Re-Docking, Cross-Docking and Ligand Enrichment in various Well-Resoluted MAO-B Receptors. Int J Pharm Sci Res 13: 1099–1107. http://dx.doi.org/10.13040/IJPSR.0975-8232.13(3).1000-12
